# Supplementary figures and images for: Optogenetics reveals Cdc42 local activation by scaffold-mediated positive feedback and Ras GTPase
Source: PLoS Biol. 2020 Jan 24;18(1):e3000600. doi: 10.1371/journal.pbio.3000600 (PMC7002011; doi:10.1371/journal.pbio.3000600)

Figure S1

A

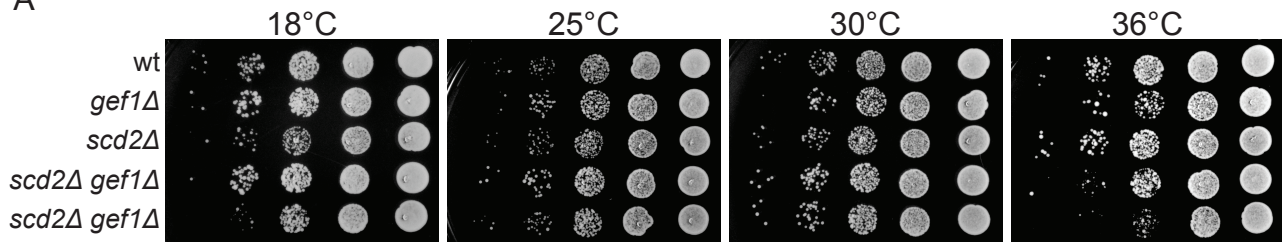

B

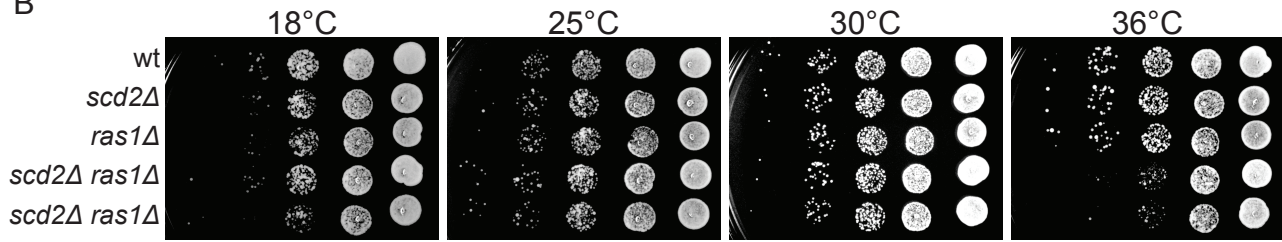

C

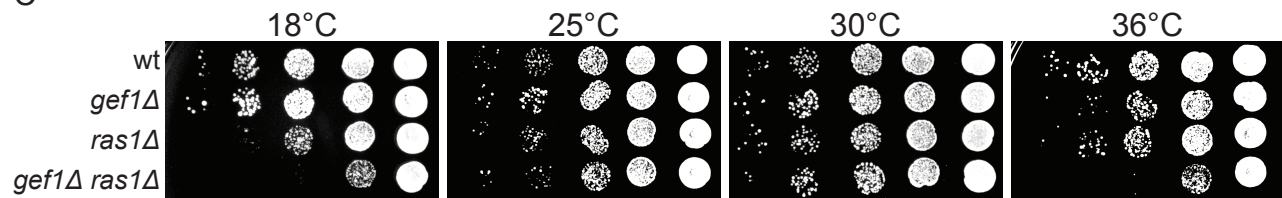

Supplement: S1 Fig — (A-C) Tenfold serial dilutions of strains with indicated genotypes spotted on YE-containing plates incubated at the specified temperatures. YE, yeast extract. (PDF) [file pbio.3000600.s001.pdf]

Figure S2

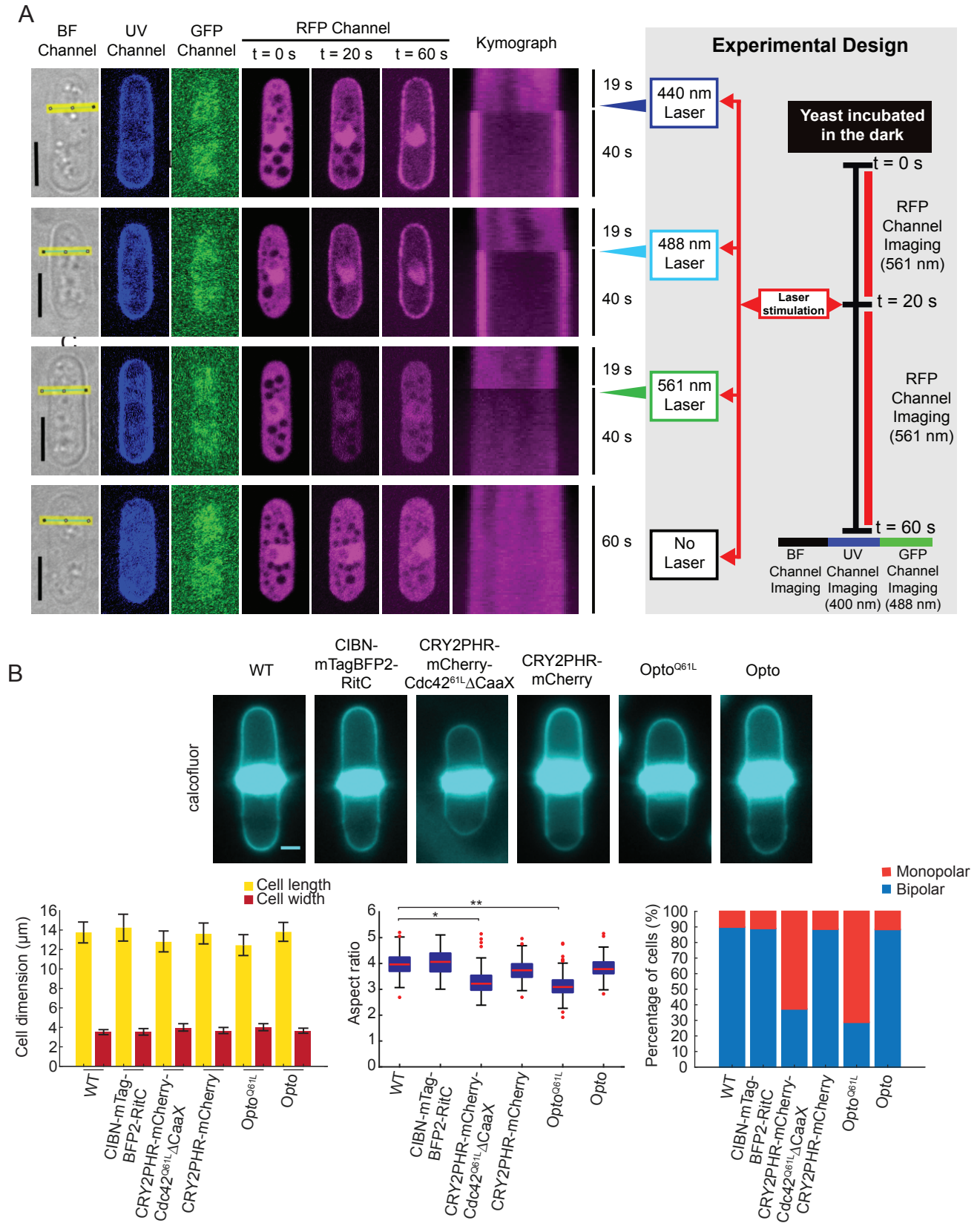

Supplement: S2 Fig — (A) Blue light–dependent cortical recruitment of CRY2PHR-mCherry in cells expressing CIBN-mTagBFP2 targeted to the plasma membrane. Scale bar = 5 μm. The scheme on the right explains the experimental design demonstrating blue-light specificity. (B) Cell length and width measurements, aspect ratio, and bipolarity of calcofluor-stained cells grown in the dark. The CRY2PHR-CIBN optogenetic system does not cause changes in cell dimensions. Cytosolic Cdc42Q61L causes moderate cell length shortening, with significant impact on the cell aspect ratio, irrespective of the presence of CIBN (pCdc42Q61L = 0.02; pOptoQ61L = 0.003 relative to wild-type cells; other comparisons yield pWTvsCIBN = 0.3; pWTvsCRYPHR-mCh = 0.1; pWTvsOpto = 0.1. Monopolar and bipolar growth were assessed on septated cells. All underlying numerical values are available in S10 Data. (PDF) [file pbio.3000600.s002.pdf]

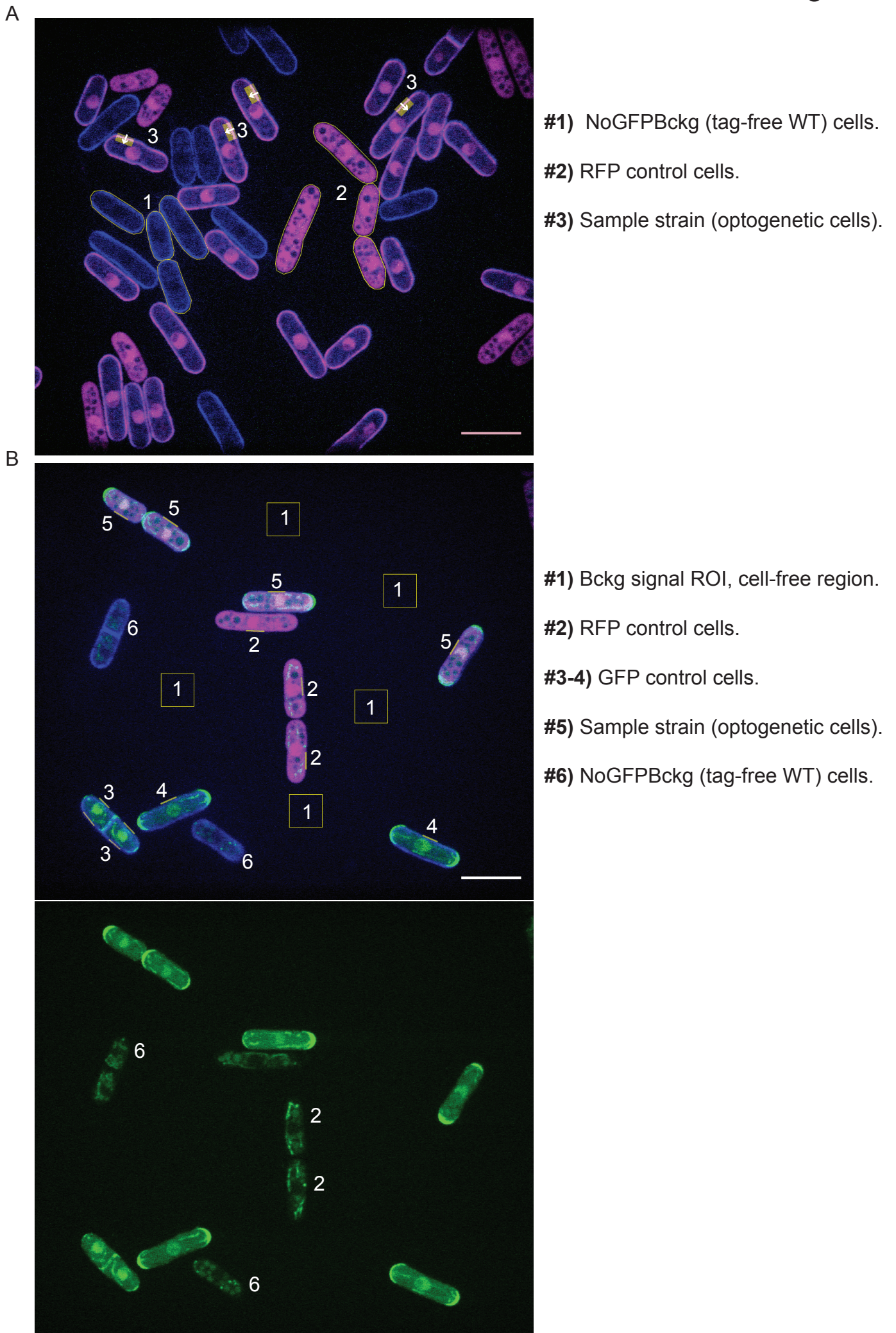

Supplement: S3 Fig — (A) Representative initial (t = 0 s) merged image of plasma-membrane recruitment dynamic experiments performed for OptoQ61L and Opto systems. Shown is the Opto system prior to stimulation with 50-ms GFP laser pulses. Cells labeled as 1 are the tag-free cells used to correct the raw data for NoGFPBckg (see Eqs 1 and 2 in Methods). Cells labeled as 2 are the RFP control cells used to calculate the RFP bleaching coefficient (see Eq 1 in Methods). Cells labeled as 3 are the optogenetic cells from which plasma-membrane recruitment dynamics were measured (Raw RFP signal parameter in Eq 2 in Methods). ROI = 15 pixels long by 36 pixels wide (roughly 1.25 μm by 3 μm). (B) (Top) Representative initial (t = 0 s) merged image of the relocalization of GFP-tagged proteins to cell sides experiments. Shown are wild-type and Opto CRIB-3GFP cells prior stimulation with blue light. ROIs labeled as 1 show the cell-free regions used to correct the raw data for Bckg (see Eqs 7, 8, and 9 in Methods). Cells labeled as 2 are RFP control cells used to calculate RFP bleaching coefficient (see Eq 7 in Methods). Cells labeled as 3–4 are GFP control cells used to calculate GFP bleaching coefficient and as control cells for cell-side relocalization of GFP-tagged endogenous proteins (see Eq 8 in Methods). Cells labeled as 5 are optogenetic cells from which cell-side relocalization of GFP-tagged endogenous proteins was monitored (see Eqs 9–13 in Methods). ROI = 3 pixels wide by 36 pixels long (≈0.25 μm by 3 μm). (Bottom) GFP channel from the merged image shown above to illustrate the background fluorescence signal in non-GFP-containing cells (labeled as 2 and 6). Bars = 10 μm. ROI, region of interest. (PDF) [file pbio.3000600.s003.pdf]

Figure S4

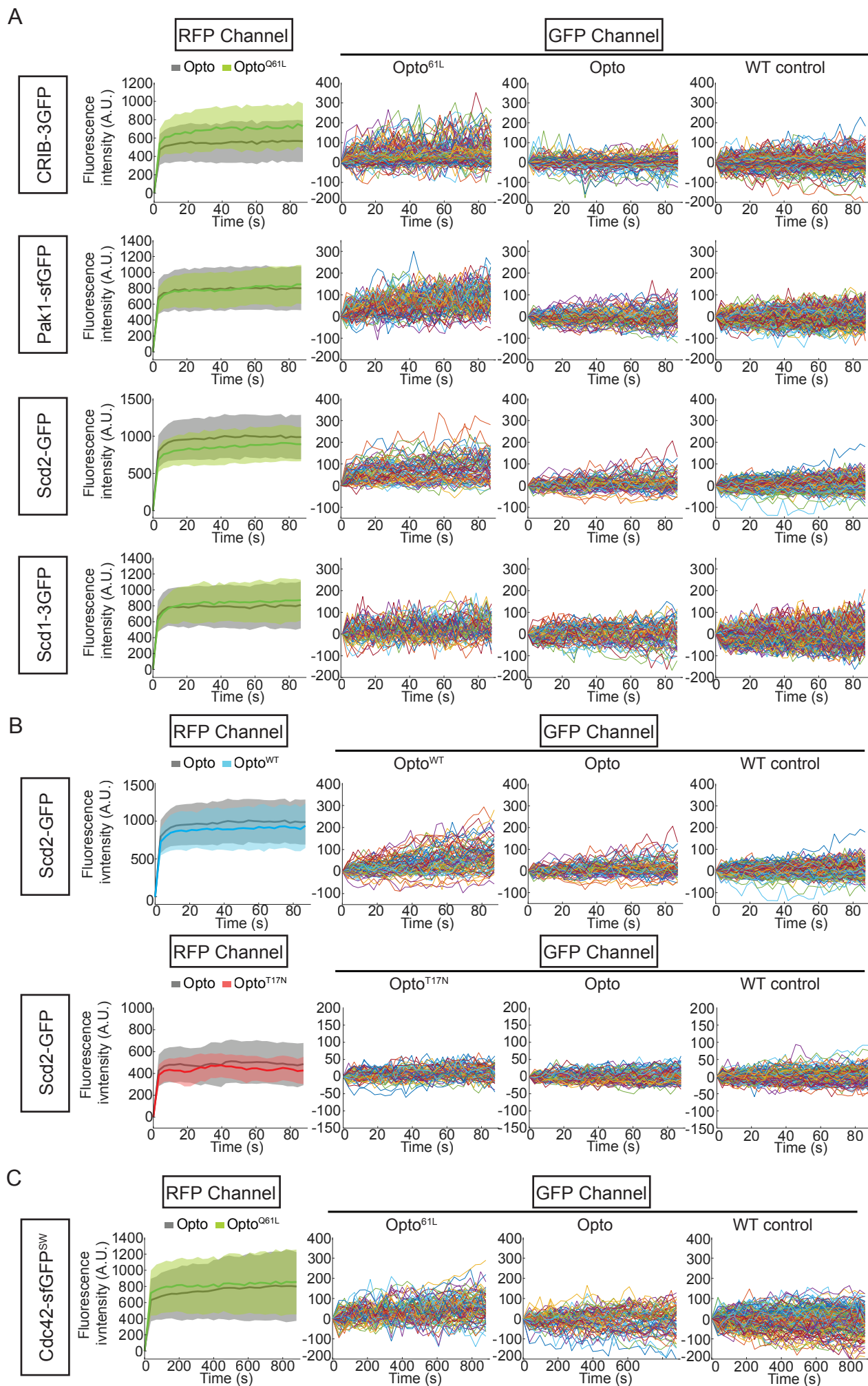

Supplement: S4 Fig — (A) Single-cell traces corresponding to the average plots shown in Fig 3C. The left column shows the average RFP signal at the plasma membrane of wild-type OptoQ61L and Opto cells. The 3 other graphs show, from left to right, single-cell GFP traces of OptoQ61L, Opto, and wild-type control cells for CRIB-3GFP, Pak1-sfGFP, Scd2-GFP, and Scd1-3GFP in otherwise wild-type cells. (B) Single-cell traces corresponding to the average plots shown in Fig 3E. The left column shows the average RFP signal at the plasma membrane of OptoWT (top) and OptoT17N (bottom) cells. The 3 other graphs show, from left to right, single-cell GFP traces of OptoWT (top) and OptoT17N (bottom), Opto, and wild-type control cells for endogenous Scd2-GFP. Note that the OptoWT and OptoQ61L experiments were performed in parallel, and thus, the Opto and wild-type control single-cell GFP traces are identical to those shown for Scd2-GFP in (A). (C) Single-cell traces corresponding to the average plots shown in Fig 3F–3G. The left column shows the average RFP signal at the plasma membrane of OptoQ61L and Opto cells. The 3 other graphs show, from left to right, single-cell GFP traces of OptoQ61L, Opto, and wild-type control cells for endogenous Cdc42-sfGFPSW. N = 3 experiments with n > 20 cells. All underlying numerical values are available in S11 Data. (PDF) [file pbio.3000600.s004.pdf]

Figure S5

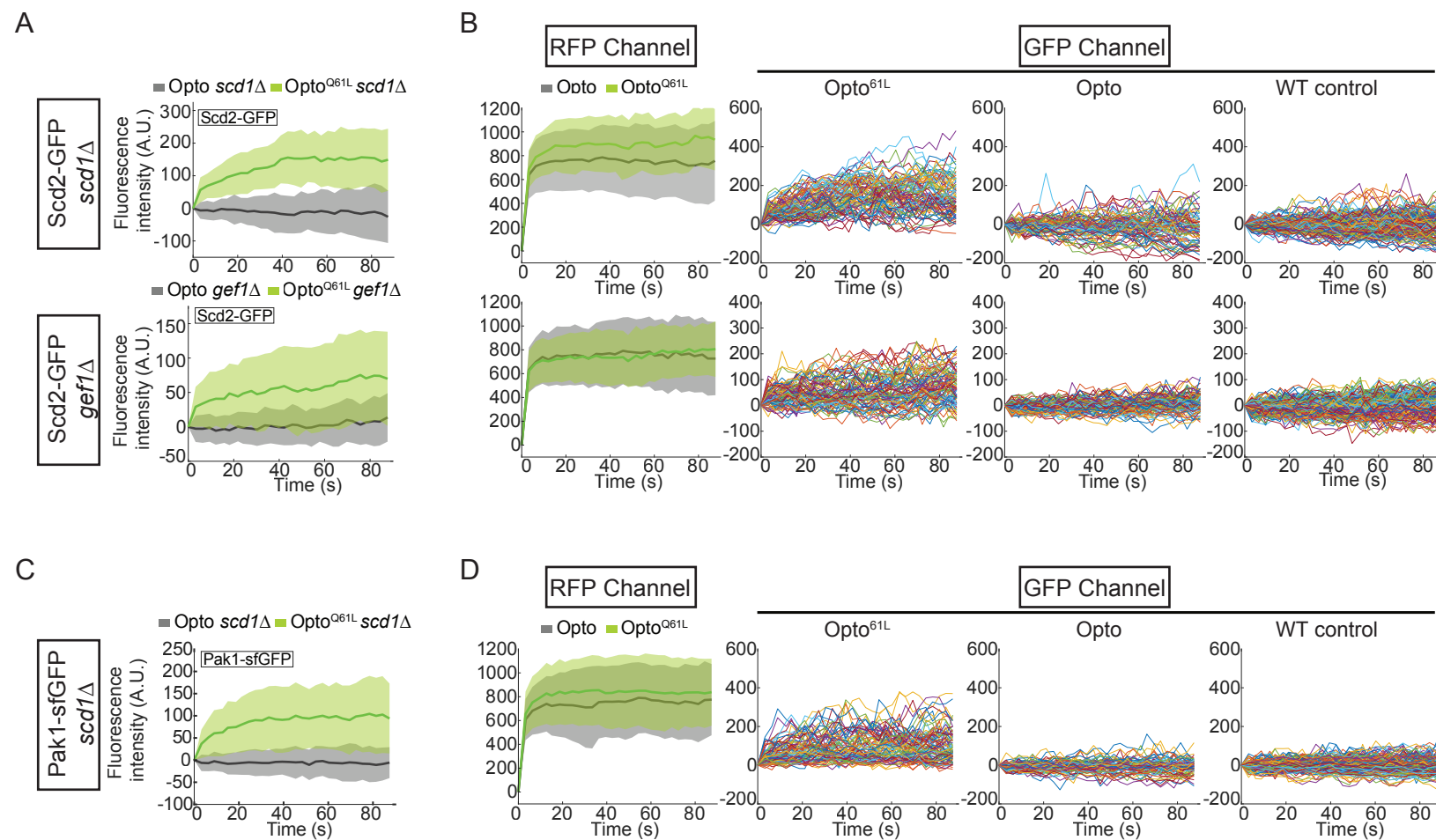

Supplement: S5 Fig — (A) OptoQ61L-induced cell-side accumulation of Scd2-GFP in scd1Δ (top) and gef1Δ (bottom) cells. N = 3; n > 20 cells per experiment; pscd1Δ = 2e-24 and pgef1Δ = 7.6e-16. (B) Single-cell traces corresponding to the average plots shown in (A). The left column shows the average RFP signal at the plasma membrane in OptoQ61L and Opto cells of the indicated genotype. The 3 other graphs show, from left to right, single-cell GFP traces of OptoQ61L, Opto, and control cells for Scd2-GFP in scd1Δ and gef1Δ cells. N = 3 experiments; n > 20 cells. (C) OptoQ61L-induced cell-side accumulation of Pak1-GFP in scd1Δ. N = 3; n > 20 cells per experiment; p = 1.3e-22. (D) Single-cell traces corresponding to the average plots shown in (C). The left column shows the average RFP signal at the plasma membrane in OptoQ61L and Opto cells of indicated genotype. The 3 other graphs show, from left to right, single-cell GFP traces of OptoQ61L, Opto, and control cells for Pak1-sfGFP in scd1Δ cells. N = 3 experiments; n > 20 cells. All underlying numerical values are available in S12 Data. (PDF) [file pbio.3000600.s005.pdf]

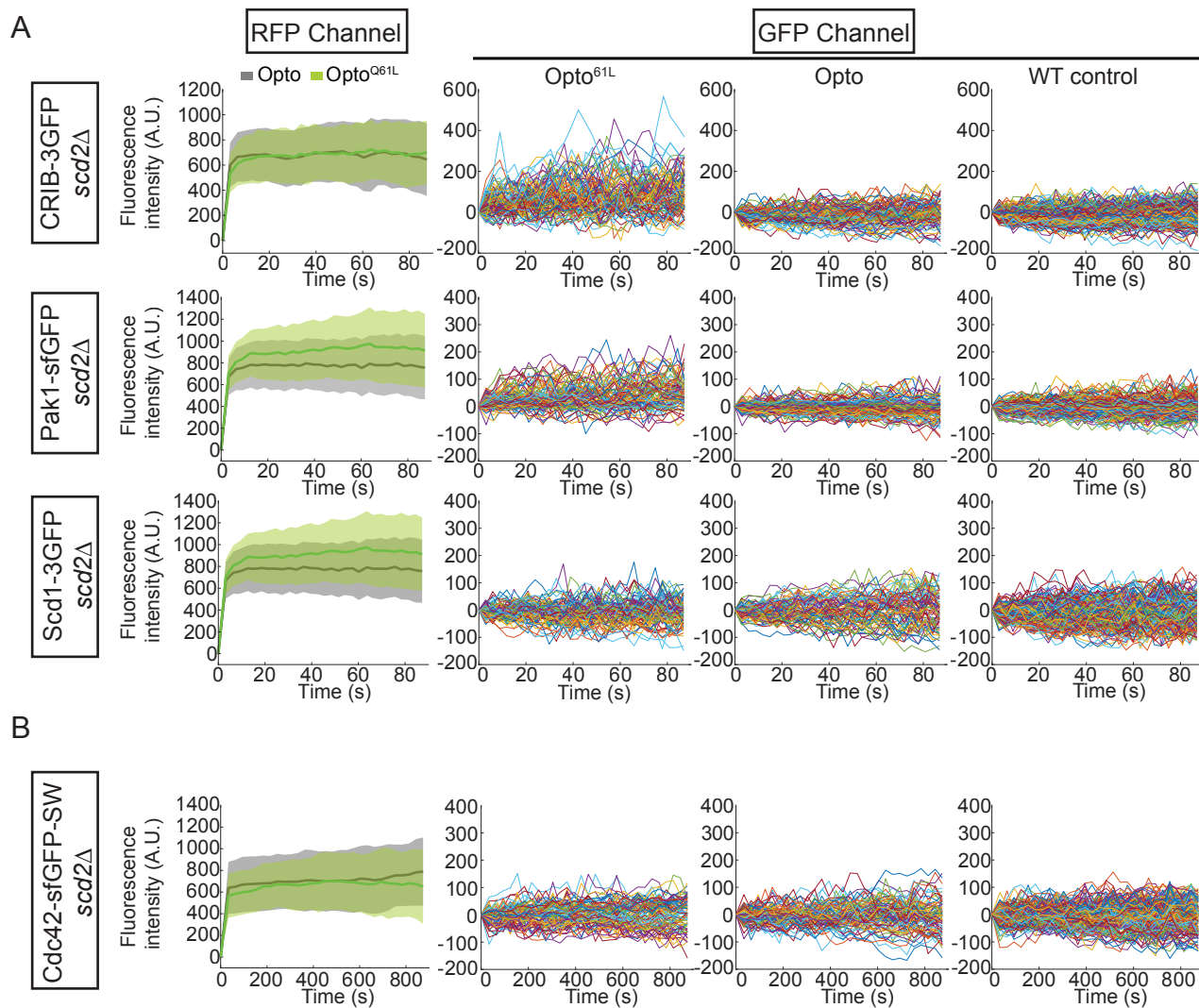

Supplement: S6 Fig — (A) Single-cell traces corresponding to the average plots shown in Fig 4A. The left column shows the average RFP signal at the plasma membrane of scd2Δ OptoQ61L and Opto cells. The 3 other graphs show, from left to right, single-cell GFP traces of OptoQ61L, Opto, and control cells for CRIB-3GFP, Pak1-sfGFP, and Scd1-3GFP in scd2Δ cells. (B) Single-cell traces corresponding to the average plots shown in Fig 4D. The left column shows the average RFP signal at the plasma membrane of scd2Δ OptoQ61L and Opto cells. The 3 other graphs show, from left to right, single-cell GFP traces of scd2Δ OptoQ61L, Opto, and wild-type control cells for endogenous Cdc42-sfGFPSW. N = 3 experiments with n > 20 cells. All underlying numerical values are available in S13 Data. (PDF) [file pbio.3000600.s006.pdf]

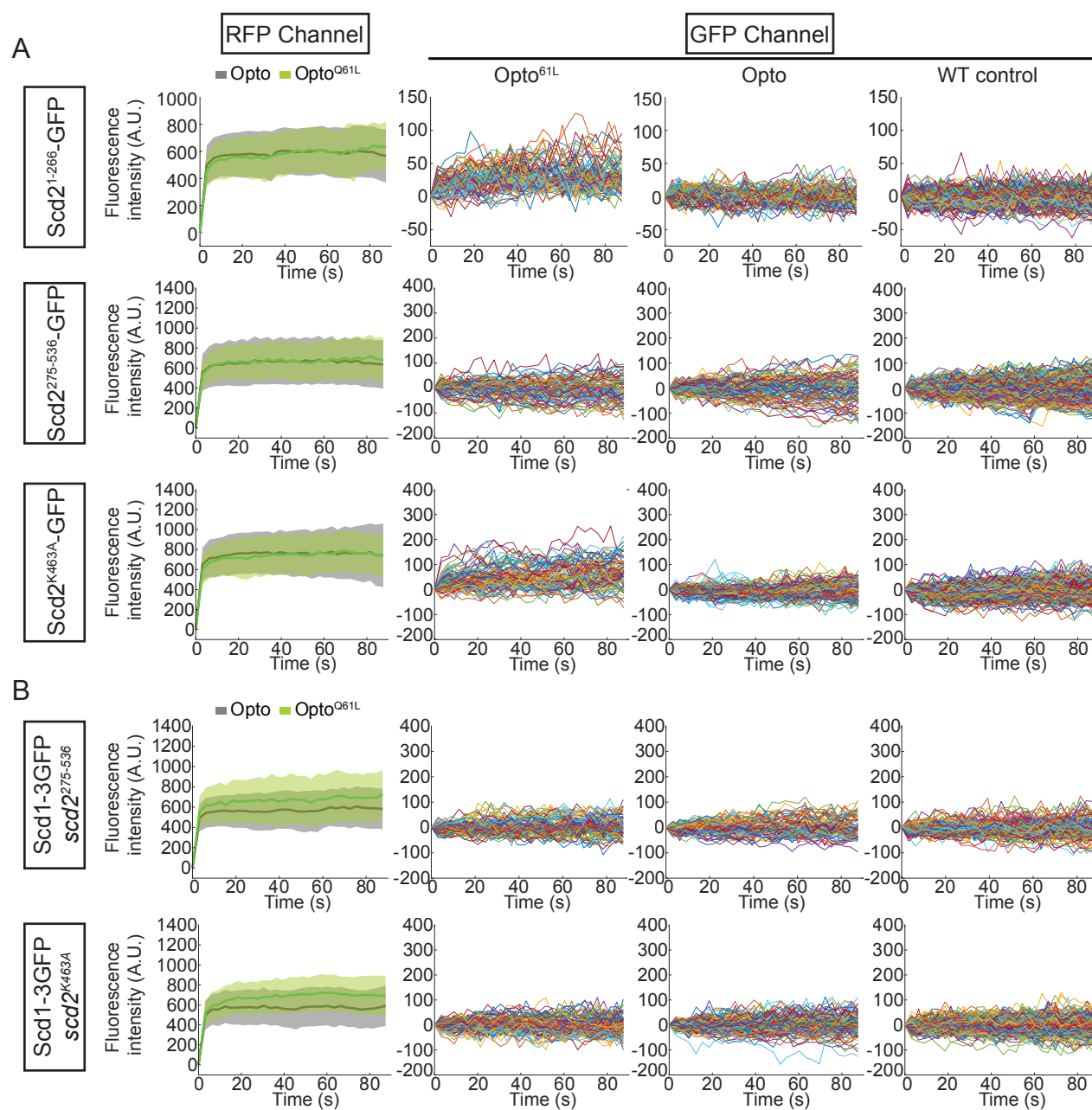

Supplement: S7 Fig — (A) Single-cell traces corresponding to the average plots shown in Fig 5D. The left column shows the average RFP signal at the plasma membrane of scd21-266, scd2275-536, and scd2k463A OptoQ61L and Opto cells. The 3 other graphs show, from left to right, single-cell GFP traces of OptoQ61L, Opto, and control cells for Scd21-266-eGFP, Scd2275-536-eGFP, and Scd2K463A-eGFP. (B) Single-cell traces corresponding to the average plots shown in Fig 5E. The left column shows the average RFP signal at the plasma membrane of scd2275-536 and scd2k463A OptoQ61L and Opto cells. The 3 other graphs show, from left to right, single-cell GFP traces of OptoQ61L, Opto, and control cells for Scd1-3GFP in scd2275-536 and scd2K463A cells. N = 3 experiments with n > 20 cells. All underlying numerical values are available in S14 Data. (PDF) [file pbio.3000600.s007.pdf]

Figure S8

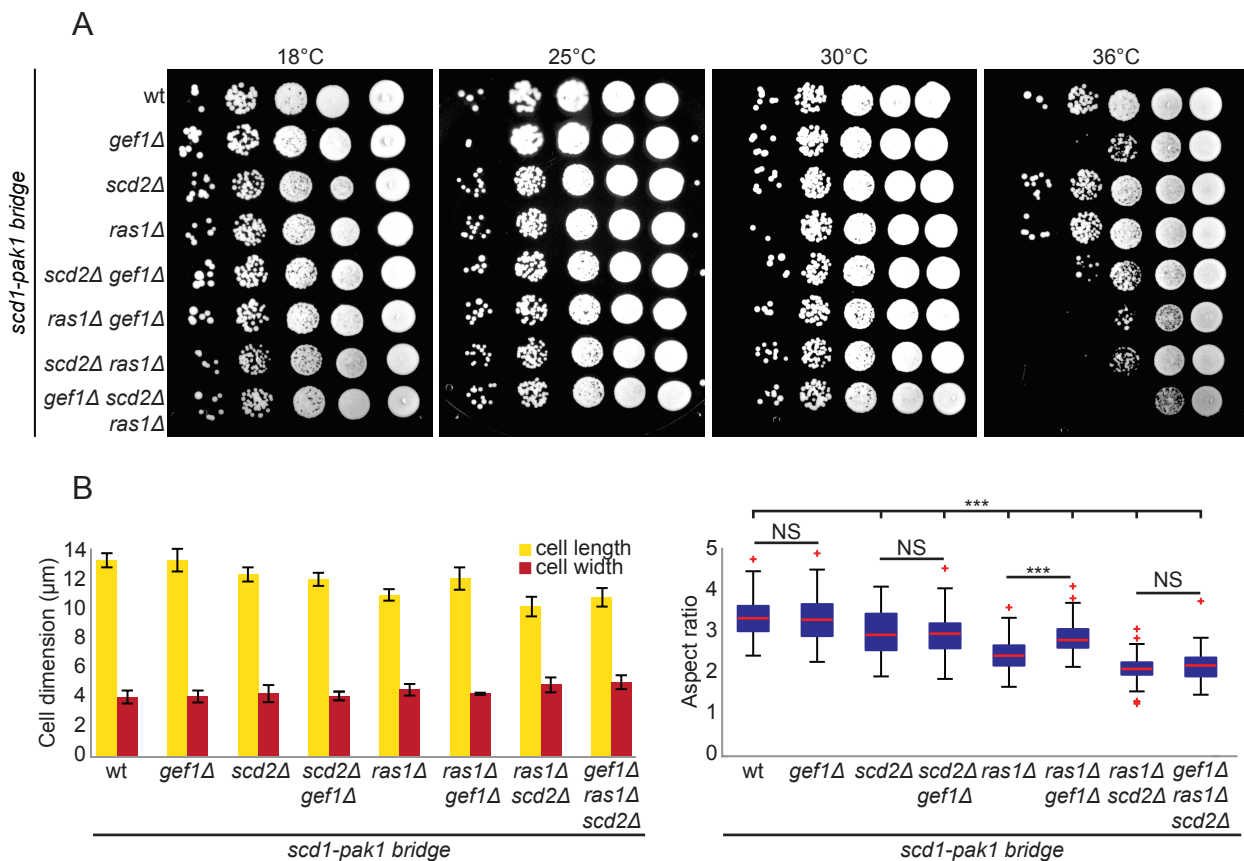

Supplement: S8 Fig — (A) Tenfold serial dilutions of strains with indicated genotypes spotted on YE-containing plates incubated at the specified temperatures. (B) Mean cell length and width at division (left), and aspect ratio (right), of strains with indicated genotypes. N = 3 experiments with n > 30 cells; ***3.5e-48 ≤ p ≤ 2e-7. Bar graph error bars show standard deviation; box plots indicate the median, 25th and 75th percentiles, and most extreme data points, not considering outliers, which are plotted individually using the red “+” symbol. All underlying numerical values are available in S15 Data. YE, yeast extract. (PDF) [file pbio.3000600.s008.pdf]

Figure S9

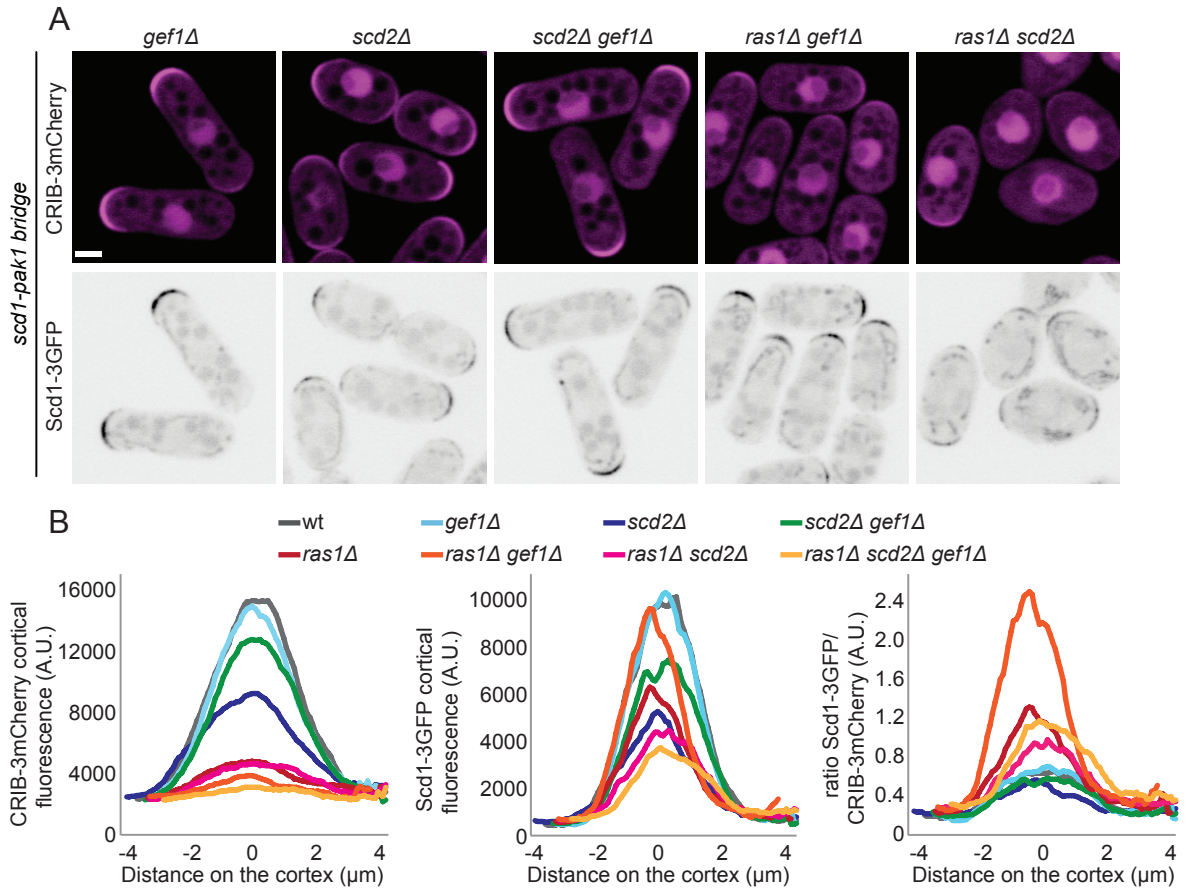

Supplement: S9 Fig — (A) Localization of Scd1-3GFP (B/W inverted images) and CRIB-3mCherry (magenta) in gef1Δ, scd2Δ, scd2Δ, gef1Δ, ras1Δ, gef1Δ, and scd2Δ, ras1Δ cells expressing the scd1-pak1 bridge. (B) Cortical tip profiles of CRIB-3mCherry (left) and Scd1-3GFP (middle) and ratio of Scd1-3GFP and CRIB-3mCherry (right) fluorescence at the cell tip of strains as in (A); n = 30 cells. Bar = 2 μm. All underlying numerical values are available in S16 Data. B/W, black and white. (PDF) [file pbio.3000600.s009.pdf]
