# Supplementary material for: Optogenetics reveals Cdc42 local activation by scaffold-mediated positive feedback and Ras GTPase
Source: PLoS Biol. 2020 Jan 24;18(1):e3000600. doi: 10.1371/journal.pbio.3000600 (PMC7002011; doi:10.1371/journal.pbio.3000600)
Supplement: Attachment — Submitted filename: Response to reviewers_191102.docx [file pbio.3000600.s033.docx]

**Response to reviewers:**

*Reviewer #1: 

This excellent paper uses optogenetic approaches to dissect the polarity control circuit in fission yeast. The major conclusions are that Ras1 acts both to localize and stimulate the Cdc42 GEF Scd1, and that the scaffold protein Scd2 acts to promote positive feedback by linking GTP-Cdc42 effector Pak1 to Scd1. These conclusions closely parallel similar conclusions about the budding yeast system, but the paper nevertheless represents a major advance for two reasons. First, it was unclear how generally the proposed polarity circuit would apply, and given the evolutionary distance between the two yeasts this is an important extension. Second, the use of cutting-edge methods cements the model by presenting findings not previously shown. The paper is also notable for the care taken to provide appropriate controls, and to conduct rigorous image analysis. Overall, this is a thorough and well-executed study on an important problem. I had a number of questions and suggestions as I
read through, with the most important being #3-4 which affect a major conclusion.*

We thank the reviewer for her/his appreciation of our work and constructive comments.

*Questions and suggestions:

1.	The Opto-42 looks patchy on the membrane in Fig. 2G as compared to Opto alone in Fig. 2B,C—any idea why? Is there a tendency to aggregate? Or a correlation (or anti-correlation) with other patchy structures like eisosomes or nodes? Or exclusion by cortical ER?*

The patchiness is present not only for Opto-42, but also for Opto alone. The key difference between panels 2G and 2B-C is the timing. Clustering of Cry2 has been described previously (Bugaj et al, Nat Methods 2013; Tasmili et al, Nat Commun 2014; Park et al, Nat Commun 2017) and is amplified with time. We have now stated this in the text in relation to the new panel 3A, which shows spatial correlation between these clusters and Scd2. We have not examined whether there was any correlation with endogenous cellular structures.

*2.	The lateral recruitment of GFP-tagged polarity proteins documented in Fig. 3A and S3 appears quite weak. While the care taken with controls and image analysis and statistics makes a convincing case that there IS recruitment, I was left wondering why it was so weak relative to the tip signal, and how such weak recruitment could lead to the longer term depolarized growth in Fig. 1?*

The reason it is weak is that we have purposefully focused on the early timepoint of recruitment to ensure we were looking at a direct effect of Cdc42 activity. Over longer times, the recruitment becomes stronger and leads to punctate distribution of polarity proteins all around the cell cortex, which explains the observed depolarized growth. To illustrate this point, we have now included a time-lapse analysis over 11h, where Scd2 is clearly recruited to side-localized Opto-Q61L and the cells start rounding up. This is now shown in new panel 3A. This time lapse also illustrates well the spatial coincidence of Scd2 with clusters of Opto-Q61L (shown in panel 3B).

*3.	The recruitment of Scd1 to cell sides by optoQ61L was especially weak, and seemed limited to a few puncta-much more so than CRIB or Scd2 signals. Do the authors have an explanation for this? Or for the internal blobs and filaments seen with this probe?*

Scd1 GEF is difficult to image (even at its natural location at cell tips in wildtype cells), likely because it is low abundance in cells, which is why we have used a 3GFP-tagged allele. The internal blobs and filaments are likely to be background fluorescence (vacuoles and mitochondria) that become apparent when imaging low-intensity probes. We have now specified this in the figure legend. We also refer the reviewer to Figure S3B, which shows a mix of GFP-labelled and unlabeled cells, where these blobs and filaments are apparent in both types of cells. We added to this figures an image of the GFP channel alone to illustrate this better. We have also tried to repeat the Scd1 recruitment experiments using a potentially brighter Scd1-mNeonGreen strain, but the signal was unfortunately weaker and less stable than that of Scd1-3GFP.

*4.	An important conclusion, that Scd2 mediates positive feedback recruitment of Scd1, was hard for me to assess. The main problems are that Scd1 recruitment was so weak even with Scd2 (see point 3) and that Scd1 signals are low in scd2 deletes, as the authors acknowledged. I’m not sure that the failure to see “competition” with tip signal gets around these issues. One potential way to make this more convincing would be to show whether Cdc42 gets recruited to cell sides by OptoQ61L in the absence of Scd2.*

We have performed the requested experiment, which is now shown in figure 4C-D: in *scd2∆* cells, endogenous Cdc42-GFP^SW^ intensity changes neither at cell tips nor at cell sides upon OptoQ61L cortical recruitment. This experiment confirms our findings that Scd2 is essential for the positive feedback.

*5.	The localization of Scd2K463A and Scd21-266 are different—why? If I understood correctly, both should bind Cdc42 and neither should bind Scd1, so why the difference? Are they expressed at different levels?*

The reviewer is right in pointing out that these two Scd2 alleles exhibit distinct localization. This is unlikely to be due to expression levels. Indeed, measurements of total GFP fluorescence for Scd2^K463A^-GFP and Scd2^1-266^-GFP strains show similar expression levels, both slightly higher than WT (now shown in figure 5B). In the *S. cerevisiae* homologue Bem1, the PX domain, which is present in the first but not the second allele, has been shown to bind phosphoinositides (Ago et al, BBRC 2001; Stahelin et al, JBC 2007). It is possible that this interaction contributes to the enhanced localization of Scd2^K463A^. We have pointed out this possibility in the manuscript.

*6.	Fig. 6 exploits the Scd1-Pak1 bridge to bypass the role of Ras1 in Scd1 recruitment and show that Ras1 increases GTP-Cdc42 at cell poles in a manner not fully explained by the level of Scd1. The authors imply/conclude (e.g. section and Figure title, as well as Discussion) that Ras1 promotes activation of Scd1.  But mechanistically, couldn’t this work by other means—like Ras1-mediated suppression of Cdc42 GAPs?*

We agree that our data did not show whether Ras1 exerted its effect on Cdc42 activation by enhancing the activity of Scd1 or through antagonizing a Cdc42 inhibitor. To differentiate between the two possibilities, we reasoned that if Ras1 acts through Scd1 to promote Cdc42 activity, *scd1∆* should be epistatic over *ras1∆*. In other words, the levels of Cdc42-GTP at cell tips should be identical in *scd1∆* single mutants and *scd1∆* *ras1∆* double mutants. By contrast, if Ras1 promotes Cdc42-GTP levels by other means, the double mutant should show reduced Cdc42-GTP relative to *scd1∆*, as it does in *scd1+* cells. Measurements of CRIB levels at cell tips of *scd1∆* single mutants and *scd1∆* *ras1∆* double mutants show identical very low values. This is in agreement with epistasis and indeed suggests that Ras1 acts through Scd1. We added this piece of evidence to figure 7E-F. In further support of this idea, in experiments performed in response to point 8 below, we also found that the broad distribution of Cdc42-GTP in cells with uniform Ras1 activity fully depend on Scd1 (please see below). Thus, we feel that the previously unsupported statement that Ras1 promotes activation of Scd1 is now supported by two independent lines of evidence.

*7.	p. 12: The authors state that scd2 gap1 mutants localize Scd1 and CRIB “all around the membrane”, citing Fig. 7D,G. But that is not what it looks like to this reader: the signals in 7D are mainly found in patches and have very different concentrations around the membrane; and 7G is not about localization of probes.*

We have rephrased the manuscript to be more precise and corrected the wrong figure reference.

*8.	Fig. 7F shows a very interesting finding that Scd1-Pak1 and Gef1-Pak1 bridge strategies yield very different outcomes in the gap1 scd2 context.  They conclude that Gef1 is not involved in positive feedback, but I don’t think this experiment addresses that. Rather, it shows an artificial bridge that SHOULD yield positive feedback by localizing Gef1 activity to sites enriched for active Cdc42 doesn’t work. Why wouldn’t Gef1-Pak1 work? Is it able to rescue scd1 gef1 mutants?*

This is an interesting question and we thank the reviewer to have incited us to look more closely at the reason for the failure of the Gef1-Pak1 bridge to restore cell shape to *gap1∆ scd2∆* cells, as this revealed interesting biology. We first tested whether Gef1 in the Gef1-Pak1 bridge is able to rescue the lethality of *scd1∆ gef1∆* mutants, and found it to be the case. In fact, we found that *scd1∆* cells expressing the Gef1-Pak1 bridge are not only viable but show better polarization than *scd1∆* single mutants, suggesting that bridging Gef1 to Pak1 enhances its activity and/or localization (Fig 8G). Therefore, Gef1 is active when bridged to Pak1.

This raises the question, as pointed out by the reviewer, of why the active Gef1-Pak1 bridge is not as potent as Scd1-Pak1 to restore cellular rod shape in *gap1∆ scd2∆* cells. We envisaged two possible explanations. First, the Gef1-Pak1 bridge may provide less efficient positive feedback than Scd1-Pak1, for instance due to differences in the intrinsic activity of Gef1 and Scd1 or other issues, such as orientation of the GEF in the complex.

Alternatively, we reasoned that Scd1, which is still present in *scd2∆ gap1∆* cells, may bias the system and prevent the Gef1-Pak1 bridge to polarize the cell. Indeed, in absence of the Scd2 scaffold, the uniform Ras1 activity in *gap1∆* cells may promote Scd1 recruitment and activation, leading to Cdc42 activation without spatial restriction. The Gef1-Pak1 bridge may thus be unable to promote polarization because of the unrestricted localization and activity of Scd1 all over the cortex. To test this possibility experimentally, we deleted *scd1* from *scd2∆ gap1∆* Gef1-Pak1 bridge cells. Remarkably, the resulting *scd2∆ gap1∆* *scd1∆* Gef1-Pak1-bridge cells were rod-shaped (similar to *scd2∆ gap1∆* Scd1-Pak1 cells). Thus, the Gef1-Pak1 bridge is perfectly able to confer positive feedback, but is unable to compete with Scd1.

The conversion of *scd2∆ gap1∆* Gef1-Pak1 cells from round to rod shape upon *scd1* deletion also supports the view that unrestrained Cdc42 activation by uniformly-localized Ras1-GTP requires Scd1 (addressing point 6 above).

We have added these new data to the manuscript in Fig 8G.

*9.	To actually address whether Gef1 is involved in positive feedback, it would be interesting to know whether Gef1 is recruited to cell sides by OptoQ61L.*

We have performed the requested experiment, but unfortunately the results are not interpretable. Gef1 localization to cell poles is very unreliable. Recent data published by the Sawin lab (Tay et al, JCS 2018) has suggested that Gef1 pole localization is actually an artefact of stress. In our experiments aimed at testing Gef1 recruitment by OptoQ61L, Gef1 was present at the cell pole only in the first timepoint of our experiments. It was not recruited to cell sides, but also disappeared from cell poles, irrespective of optogenetic activation. We thus cannot make any firm conclusion about its possible recruitment by OptoQ61L.

10.	Fig. 7H,I: This experiment introduces a new reagent Scd2-CAAX but does not validate that it really localizes all around the membrane: such validation is needed to interpret the experiment.

We have investigated the localization of Scd2-CAAX by tagging it with GFP. In wildtype cells, Scd2-CAAX, like Scd2^WT^, is enriched at cell tips, but in addition can be detected elsewhere around the cell’s plasma membrane. This localization is consistent with Scd2-CAAX still responding to localization cues of Scd2^WT^ and showing an extended residency at the membrane. This localization is now shown in Fig 9A.

We have also consolidated this finding by linking Scd2 to the plasma membrane with an amphipathic helix (RitC), which yields very similar phenotypes of both extended decoration of the cell cortex and wider cells when Ras is active uniformly at the cortex in *gap1∆*. These data are now shown in Fig 9C-D.

*Issues with presentation:

1.	P-values are provided in Figure Legends, but there was no description or justification of what statistical test was applied where.*

We apologize that the paragraph on statistical analysis was absent from the methods. This has now been corrected. We used either Kruskal-Wallis or Student T-test, as described in the last paragraph of the methods.

*2.	It would be good to see images of Cdc42 recruitment (as in Fig. 3A) to back up the quantification provided in Fig. 3C. Unlike for the other probes, one would expect to see a basal level of Cdc42 at the membrane.*

We now provide the images in Fig 3H. As stated by the reviewer, endogenous Cdc42 is already at the lateral plasma membrane in WT cells, which makes visual evaluation of its recruitment difficult. To enhance the visualization, we provide two complementary views of the data: a fluorescence time lapse comparing mixed OptoQ61L and control cells, which clearly show the loss of Cdc42 from cell poles only in the OptoQ61L cells, and the same images from which the fluorescence signal from t0 has been subtracted. Any region showing a signal thus represents an increase over t0: the OptoQ61L cells show signal on cell sides and none at the cell tips, illustrating the increase of Cdc42 at cell sides. The control cells show signal only at cell tips (which is not really increasing but arises due to signal displacement from the subtracted t0 cell tip upon cell growth).

*3.	The authors have taken the excellent approach of including negative control cells mixed in with the cells of interest on the same imaging slab, which eliminates potential day-to-day and slab-to-slab differences as a basis for their observations. While this is explained in the methods, it would be beneficial for the average reader to include a mention in the Figure Legends or main text. This would alert the reader to the significance of the BFP-only and RFP-only cells in Fig. 2B, for example.*

We have now added mention of this in the text.

*4.	The use of inverted black on white images is excellent and allows the reader to optimally appreciate the results. And the signal from optogenetic system constructs is so strong that color panels are OK there, but why use the much less clear magenta-on-black for various panels in Fig. 5-7?*

We agree that the contrasting on the magenta/black panels is not as good as on the B/W ones. However, we have made the choice of using two different coloring schemes to best illustrate that different fluorescent proteins are shown in the same cells (as opposed to the same genetic background but different cells such as in Fig 8D). We feel that the use of B/W throughout would not make this point as clearly.

5.	p. 34 top: Should “cell side tip” be “cell tip”?

Thanks for spotting this typo, which is now corrected.

*--

Reviewer #2: 

This manuscript examines the mechanisms of Cdc42 activation in fission yeast S. pombe. The authors use both conventional genetics and optogenetics to dissect the Scd1/Scd2/Pak1 polarity complex. The optogenetic studies indicate that membrane recruitment of an activated form Cdc42 appears to be able to recruit components Scd1, Scd2, and Pak1 and that that Scd1 recruitment appears to require Scd2. The authors claim direct recruitment of Scd2 by Cdc42, however these results are not shown in full and what is shown is not fully compelling. Overall the results deepen the parallels between the behavior of these Cdc42 regulators in budding and fission yeast. The strongest part of the manuscript are the conventional cell biological and genetic studies that demonstrate that Ras1 plays a role in both recruitment and activation of Scd1.*

We thank the reviewer for appreciating our cell biological and genetic studies. We hope that our responses below will also help convey the strength of our efforts in optogenetics.

*The authors use the Cry2/Cib system to recruit an activated allele of Cdc42 lacking its CaaX box. The authors appear to imply that this recruited Cdc42 is normally active. However, this assumption is not well founded as this mode of tethering may not result in a Cdc42 molecule in its normal proximity, and perhaps orientation, relative to the plasma membrane. While the tagged molecule is robustly, uniformly, and rapidly recruited to the cortex, the recruitment of polarity proteins is far slower, far less robust, and highly patchy, suggesting that only a small proportion of recruited molecules are functional. There is no mention in the text of the low degree of spatial correlation between the recruited Cdc42 and its interacting partners. The recruited Cdc42 does have some biological activity as the cells exhibit light dependent depolarization. However, this could be due to either a gain of function effect (global Cdc42 activation) or a dominant negative effect (sequestering
and inactivating polarity proteins) or a combination thereof; it is not clear which is correct. However, the authors state, "This transition from rod to round shape is a clear evidence of isotropic growth triggered by the recruitment of Cdc42 activity to the plasma membrane in a light-dependent manner." The temporal resolution of the movie provided (S2) (1 hour intervals) and the absence of a marker for the growing end limits the utility of this movie. I would not assess the evidence as “clear”.*

We are fully aware that Cdc42-Q61L recruited to the membrane through the Cry2-CIBN system is not a “normally active” Cdc42. It is quite possible, even likely, that it exhibits less activity than WT Cdc42-GTP. We know however that Cdc42 exhibits substantial activity when tethered to the plasma membrane in unusual ways. We had for instance shown that tethering through an amphipathic domain as used here or even a transmembrane domain yielded Cdc42 alleles able to sustain symmetry-breaking, even if dynamics was substantially slower (see Bendezu et al, PLoS Biol 2015). In addition, as the reviewer points out, the recruited Cdc42-Q61L is active, as it triggers isotropic growth only when recruited to the plasma membrane (not in the dark) and promotes the immediate recruitment of effectors. We documented some dominant-negative effect through the sequestration of Scd2 in the nucleus also in the dark and how now explicitly stated in the text that the Opto-Q61L is not normally active. We feel that we have been transparent in our assessment of this reagent, which we have used as a tool to acutely direct Cdc42 activity to cell sides.

To clarify the issue raised about the long-term time lapse, we have now performed long time lapse experiments with shorter imaging interval showing both the re-localization of Scd2 away from the cell pole and the rounding of cells (shown in Fig 3A). These show that cell tip localization of Scd2 is lost as cells round up. We have also used this new time-lapse to illustrate the spatial colocalization of Scd2 with OptoQ61L (shown in Fig 3B).

*In addition, it is not apparent that the polarity complexes that are recruited in a Cdc42-dependent manner are highly active as the amount of CRIB-GFP recruited to the cortex is similar in the presence and absence of Scd2 (compare Fig 3A vs 4A). A possible explanation is that the recruited active Cdc42 does not recruit a particularly active GEF.*

CRIB-GFP is recruited to cell sides in both WT and *scd2∆* because CIRB binds constitutively active Cdc42Q61L, which is similarly recruited by optogenetics in both strains. It simply serves as a positive control for the optogenetic recruitment of Cdc42 activity. Given the large amount of Cdc42-Q61L recruited by optogenetics and the fact that Cdc42-Q61L is constitutively active, whereas the amount of endogenous activated Cdc42 will be low at these early timepoints and only transiently activated (due to the presence of GAPs), CRIB-GFP is likely to principally label Cdc42-Q61L. Thus, we do not find it surprising that the amount of CRIB-GFP is similar in WT and *scd2∆* cells.

*The authors state that the recruited Cdc42 demonstrates positive feedback which had not been shown previously. Strictly speaking this claim is valid as some endogenous Cdc42 is recruited to cell sides (fig 4C), though only the quantification and not the raw imaging data are shown for this conclusion. Overall, It is difficult to make this claim in the absence of evidence showing proper light dependent polarization, as was shown with the Scd1/Pak1 bridged molecule.*

As stated in answer to reviewer 1 above, we now provide the images of endogenous Cdc42 recruitment in Fig 3H. We also note that our finding that Cdc42-Q61L recruits its own Scd1 GEF activator is also evidence of positive feedback.

*In this vein, the authors state, that prior experiments in budding yeast did not "did not directly test whether Cdc42 promotes its own activation." Again, formally this is correct, but the aforementioned paper did demonstrate that the ability of the Cdc24 GEF to induce cell polarization required its GEF activity, but not the ability to bind Bem1, implying that active Cdc42 is required for positive feedback. Overall, the authors do not summarize the more salient results of this paper. They focus instead on the cell cycle control aspects which are not particularly relevant to the present paper and don't mention the direct demonstration of light directed polarization and positive feedback among polarity proteins or the winner take all behavior of the cell even in the presence of two distinct foci of active Cdc42, which are far more applicable to this manuscript.*

The central elements in the excellent Witte et al, 2017 eLife paper that show positive feedback are the observations that optogenetic recruitment of Bem1 leads to local recruitment of endogenous Bem1, and similarly that optogenetic recruitment of Cdc24, but not inactive Cdc24, lead to local recruitment of endogenous Cdc24. Assuming that the Cdc42-PAK-Bem1-GEF complex is strictly composed of 1 copy of each protein, then this indeed shows positive feedback. The difficulty with these experiments (and the reason we did not detail them further in our first submission) is that 1) while the Bem1 experiment suggests scaffold-mediated feedback, it did not directly test whether this depended on Cdc42 activity, and 2) the Cdc24 experiment promoted Cdc24 recruitment at earlier stages than Bem1 recruitment and thus led to the interpretation that this is a different feedback independent of the scaffold. We have expanded reference to this paper in the revised submission to state that the results are also consistent with positive feedback.

*The authors stated they were unable to use their optogenetic system to perform local recruitment assays which precludes its use to address many interesting questions related to the oscillation of Cdc42 activation and even to test whether local recruitment is sufficient to induce cell polarization. It is possible that illumination with excessive levels of light resulted in global activation. The high rates of diffusion in yeast cells does not preclude the generation of a local pool of activated Cdc42.*

The issue of the Cry2/CIBN system with local recruitment is that the photo-sensitive moiety is cytosolic. In the cytosol, diffusion is fast, such that even local photo-activation will yield a pool of photo-activated molecules that populate the entire cell. We have performed these experiments and indeed observed that upon local photoactivation, Opto-Q61L was recruited to the cortex but not specifically at the site of light activation. The problem is not due to excessive levels of light leading to global activation. As stated in the manuscript, we were unfortunately unable to generate a functional system when Cry2 was anchored at the plasma membrane (likely due to the clustering properties of Cry2), which would be the way to generate a system that can be locally activated, because lateral diffusion at the plasma membrane is orders of magnitude slower than in cytosol.

Related to figure 4, the recruitment of Scd2 1-266 is a key result, but again, the raw data not shown. The authors conclude, "This suggests Cdc42-GTP interaction is a major contributor to Scd2 localization." and "Thus, Scd2 is the scaffold that promotes positive feedback by linking Cdc42-GTP to its GEF Scd1." While this conclusion is consistent with the data with the Scd2 fragments, it's relevance to the full length protein is less clear. With the full length proteins, it is possible that Cdc42 recruits Scd1 via Cdc42 > Pak1 > Scd2 > Scd1, an interpretation in line with the demonstrated functionality of the Pak1-Scd1 bridge constructs. The role of the Cdc42 binding sites in Scd2 are interesting but not well established in vivo. If the authors want to claim the Cdc42 play an important role in directly recruiting Scd2, they should compare the recruitment the full length protein in the presence and absence of a point mutations that disrupt the Cdc42 binding site. This figure would be improved with a diagram of Scd2 with domains indicated.

We have added to Fig 5D the recruitment kinetics of Scd2(1-266) by OptoQ61L. We also added a diagram of Scd2 to explain the various alleles used (in Fig 5A). We agree with the reviewer that the role of Cdc42-GTP in the recruitment of full-length Scd2 is not clearly established and have acknowledged it in the text. We unfortunately do not have a point mutant that specifically disrupts Cdc42 binding. It is thus possible that both Cdc42-GTP direct binding and binding through Pak1 contribute to Scd2 recruitment.

Overall, while I appreciate the efforts the authors have made to adapt optogenetic approaches to this problem, the results they have obtained are not as compelling as they are described. Perhaps this is due to the fact that an activated allele of Cdc42 lacking its lipid tail is not an ideal probe. Cortical recruitment of many effectors of Cdc42 requires the ability to bind to both the GTPase and the membrane, certainly there are steric limitations to these interactions. Further, as the authors are acutely aware (Bendezu, 2015), tagging small GTPases is fraught with challenges. This is a case where the best approach might be to present the data along with its limitations and limit the scope of the conclusions to those that can be unambiguously drawn from the data presented.

We have added to both results and discussion description of the limitations of use of a constitutively active Cdc42 allele artificially recruited to the plasma membrane.

*The experiments with the Scd1-Pak bridge molecule, revealing a role for Gef1 in enhancing cell polarity and those pertaining to Ras1 are more compelling. However, a number of points related to these experiments require clarification or modification.

1 - Is the function of the "bridge" dependent upon the triple GFP tag on Scd1? This construct would be predicted to form a Scd1-(Pak1)(Pak1)(Pak1) molecule which may function differently than a Scd1-(Pak1) or, indeed, the Scd1-Scd2-Pak1 endogenous complex.*

This is an interesting comment, in response of which we have performed equivalent experiments using Scd1-GFP (instead of Scd1-3GFP) to form a bridge with Pak1-GBP. This 1x-Scd1-Pak1 bridge was able to rescue *scd2∆* cells shape and yielded viable *scd2∆ gef1∆ ras1∆* triple mutant cells. We noted however that the triple mutants + 1x-bridge were less well polarized than the triple mutants + 3x-bridge. Thus, our conclusion that Scd2 provides a bridge between Scd1 and Pak1 is valid independently of 1 or 3GFP tagging, but there is some effect of valency and/or strength of interaction in enhancing the effect of the bridge. It is of course difficult to predict which bridge better mimics the WT situation. For instance, one of the three GFPs in the 3x-GFP bridge may provide a better orientation for Pak1-GBP binding than the sole one in the 1x-GFP bridge, but the data suggest that amplification of the Pak1 positional signal promotes cell polarization. We now show this new data in tables 1 and S1 and have included a description in the results and in the discussion.

*2- page 12 "Strikingly, scd2Δ gap1Δ double mutants were almost completely round and localized Scd1 and CRIB all around the membrane (Fig 7D, G)." This is not an accurate description of the images in fig 7D, particularly the second half of the sentence.*

We have re-worded the manuscript to describe this result more precisely.

*3- page 12, " These data strongly indicate that Ras1 promotes not only Scd1 localization but also its activity towards Cdc42. Thus, Ras1 acts to modulate the strength of the positive feedback." In one interpretation, this make sense, if it were a linear pathway in which Scd1 recruitment lead to CRIB recruitment. However, it is not linear, there is positive feedback, so if Ras1 only activated Scd1, then Ras1 mutants would be expected to have less Scd1 due to a reduction in positive feedback.*

We agree with the reviewer that the pathway is not linear due to the positive feedback. Therefore, a reduction in Scd1 and/or Cdc42 activity will cause a reduction in the localization of Scd1 at cell poles. This is generally what is observed, as there is a good correlation between Scd1 levels and Cdc42-GTP levels at the cell poles. We had previously made similar observations between Cdc42 protein levels and Cdc42-GTP levels (see Bendezu et al, PLoS Biol 2015). The point we are making is that *ras1∆* falls out of this general correlation and *ras1∆* mutants exhibit significantly less Cdc42-GTP (CRIB) for similar Scd1 levels. We interpret this to mean that Ras1 promotes Cdc42 activation independently of the feedback. Because Ras1-dependent Cdc42 activation is dependent on *scd1* (see the new data in response to reviewer 1 – points 6 and 8, presented in figures 7E-F and 8G), Ras1 is likely to promote Scd1 activity towards Cdc42. Therefore, it modulates the strength of the positive feedback.

The argument that Ras1 also conveys a positional signal comes from data presented in figures 1 and 8: in *scd2∆* cells that lack positive feedback, Ras1, which is active at cell poles, has a significant effect in permitting residual Scd1 localization and positioning zones of Cdc42-GTP at cell poles to promote the rod shape. Similarly, when Ras1 is active uniformly at the cell cortex in *gap1∆ scd2∆* cells, this leads to localization of Scd1 over large portions of the cell periphery. This suggests that Ras1 does not only activate Scd1, but also has effects on its localization (which could be a consequence of activation – we have not directly investigated this point).

We have modified the text to make this logic clearer.

*4- Overall the Ras1 data suggest that Ras1 primarily promotes positive feedback and has far less of an instructive role in the positioning. In the absence of Ras1 or in the absence of Gap1, the polarity complex retains the ability to localize properly. The evidence for a positional role for Ras1 far less compelling.*

The role of Ras1 in providing a positional signal is not critical in wildtype cells, likely because it is redundant with a similar role played by positive feedback components. Indeed, as mentioned in response to the previous comment, Ras1 plays a clear positional role in cells lacking Scd2: in *scd2∆* cells that do not exhibit positive feedback, residual Scd1 localization, as well as the ability of cells to form a polarized zone of growth and a rod shape, depends on Ras1; in *scd2∆ gap1∆* cells, where Ras1 is active over the entire cell periphery, this directs Scd1 away from cell poles and leads to the formation of round cells.

*minor points
page 5 "Consistently" is used incorrectly.*

Corrected.

*page 10 "Scd2K463A was "efficiently" recruited to cell sides by OptoQ61L, but was unable to induce the cell side re-localization of Scd1 (Fig 4E, S4B)". Efficiently?*

Scd2K463A is recruited by OptoQ61L just as strongly as WT Scd2 (please compare Fig 5D and 3C).

*Overall the authors over use adjectives: clearly, efficiently, strongly, etc. Let the data speak for themselves.*

We have now made a more sparing use of adverbs.

*--

Reviewer #3: 

In this Manuscript the authors have developed and used the state-of-the-art optogenetic approach to investigate regulation of Cdc42 during polarization in fission yeast cells. This is the first example of such an approach being used in fission yeast. This powerful approach has the potential to significantly advance the field of cell polarization and reveal previously unidentified or poorly understood mechanism of Cdc42 activation. Indeed, this research shows for the first time in vivo evidence of a positive feedback Cdc42 activation. Moreover, the use of a Scd1-Pak1 bridge reveal some unexpected results that bring to question our previous assumptions about the mechanism of feedback pathways involved in Cdc42 regulation. While overall the study is high quality, the data clearly presented and very well suited for PLOS Biology, the authors have not considered all potential explanations for some of their observations. This leads to some strong statements without sufficient
evidence to support them. The authors should consider alternate hypotheses for their observations and either resolve them or at the very least discuss them before the manuscript is accepted. Addition of a few controls may also help the manuscript as is detailed below.*

We thank the reviewer for her/his appreciation of our work and constructive comments.

*Major points
1.	Pak1 kinase is involved in negative feedback regulation of Cdc42. Previous work from Lew lab and McCusker labs have shown that Pak1 phosphorylates the GEF Cdc24 to diminish GEF activity. One proposed explanation for this inhibition is that Pak1-mediated phosphorylation of Cdc24 prevents interaction with the scaffold Bem1 and in the absence of Bem1 interaction Cdc24 does not localize and cannot activate Cdc42. Here the authors made a Scd1-Pak1 bridge eliminating the need for Scd2 and this leads to localization of Scd1 and Cdc42 activation. However, with the Pak1 CRIB alone or with a kinase dead Pak1 they do not see establishment of a positive feedback or cell polarization even though Scd1 is localized to the cortex. The authors claim that this indicates that Pak1 is needed for Scd1 mediated Cdc42 activation and the positive feedback. However, this is not true since pak1 mutants (orb2-34, kinase dead and switch off) all display the same phenotype of monopolar cells with hyperactivation of Cdc42 and increased Scd1 at the single growing pole. This is likely due to lack of a negative feedback. These previous observations by multiple groups suggest that pak1 is not required for Scd1 mediated Cdc42 activation and in fact absence of Pak1 further enhances this activation. In figure 5E and F, the cells appear to show significant Scd1-3xGFP at the cortex but this is spread over a larger area. Such an observation could also be explained by the lack of Pak1 mediated phosphorylation of Scd1, where Scd1 does not accumulate at local regions and thus the cells show increased cell width. What does the CRIB signal look like in these cells? It is entirely possible that Pak1 phosphorylates Scd1 only when they are in a complex and this phosphorylation tunes Cdc42 activation. In such a scenario the wild type Pak1 present in these cells will not associate with Scd1 (especially since Scd2 is absent).*

This is an interesting comment. The role of Pak1 kinase activity has indeed been attributed to negative feedback in previous work, with the idea that the kinase limits the activity of the GEF by phosphorylating it. It is possible that the reduced ability of Pak1N and Pak1-KD bridges to restore the rod shape reflects some negative function of Pak1 kinase activity in the system, although we do not have a simple idea for how it would generate the observed phenotype. We have tried to visualize CRIB in the strains in figures 6E and 6F. To this aim, we generated a CRIB-mTagBFP2 probe (to introduce a third fluorescence channel in these already complex genetic backgrounds, and hoping it would not interfere with GBP binding). In WT cells, this probe labels cell poles as well as the GFP version. However, in the triple *gef1∆ scd2∆ ras1∆* mutants, whether rescued by full-length Pak1-Scd1 bridge or truncated versions, we could not reliably detect CRIB-mTagBFP2 signal at the cell cortex. We were thus unable to determine whether the rescue with the truncated bridges lead to enhanced Cdc42-GTP levels, as would be predicted from a loss of negative feedback. We modified the manuscript to clearly state that our data indicate an important role of the Pak1 kinase domain, but leaving open the mechanism of action and mentioning the possibility of a negative role.

*2.	Another unexpected and fairly interesting result is that the Gef1-Pak1 bridge does not act the same way as the Scd1-Pak1 bridge. The authors claim that this indicates that Scd1 is the necessary for positive feedback. In figure 7 F the authors show Gef1-Pak1 bridge does not restore polarity in scd1Δgap1Δ mutants. It is not clear how Gef1 localizes to the cortex in scd2Δgap1Δ mutants without the bridge. This control would explain if interaction with Pak1 impairs Gef1 activity. Could it be that Gef1 localization or activity is impaired upon forced Pak1 interaction? Would a Gef1 bridged with the Pak1 CRIB domain also show the same phenotype. Would a constitutively localized gef1S112A mutation (Das et al 2015) when bridged to Pak1 also show the same phenotype?*

We thank the reviewer for this comment, which is similar to point 8 of reviewer 1. We looked at Gef1 localization in *gap1∆ scd2∆* without the bridge, which showed Gef1 at the cell cortex (similar to Scd1 and CRIB). We have added this data to Fig 8D. To address whether forced interaction of Gef1 with Pak1 impairs its activity/localization, we have tested whether Gef1-3GFP is able to sustain growth in *scd1∆* cells in presence of Pak1-GBP. The Gef1-Pak1 bridge was not lethal in *scd1∆* and in fact these cells showed better polarization than *scd1∆* single mutants, suggesting that, if anything, bridging Gef1 to Pak1 enhances its activity and/or localization (data added to figure 8G). We conclude that Gef1 is active when bridged to Pak1.

As also explained in response to reviewer 1, this raises the question of why the active Gef1-Pak1 bridge is not as potent as Scd1-Pak1 to restore cellular rod shape in *gap1∆ scd2∆* cells. We envisaged two possible explanations. First, the Gef1-Pak1 bridge may provide less efficient positive feedback than Scd1-Pak1, for instance due to differences in the intrinsic activity of Gef1 and Scd1 or to more trivial issues, such as orientation of the GEF in the complex. Alternatively, we reasoned that Scd1, which is still present in *scd2∆ gap1∆* cells, may bias the system and prevent the Gef1-Pak1 bridge to polarize the cell. Indeed, in absence of the Scd2 scaffold, the uniform Ras1 activity in these cells may promote Scd1 recruitment and activation, leading to Cdc42 activation without spatial restriction. The Gef1-Pak1 bridge may thus be unable to promote polarization because of the unrestricted localization and activity of Scd1 all over the cortex. To test this possibility experimentally, we deleted *scd1* from *scd2∆ gap1∆* Gef1-Pak1 bridge cells. Remarkably, the resulting *scd2∆ gap1∆* *scd1∆* Gef1-Pak1 cells were rod-shaped (similar to *scd2∆ gap1∆* Scd1-Pak1 cells). Thus, the Gef1-Pak1 bridge is perfectly able to confer positive feedback, but is unable to compete with Scd1. This data is shown in Fig 8G.

Given these results, we have not tested a Gef1 bridge to Pak1 N-terminus alone, or a Gef1S112A.

*Minor points
1.	Throughout the paper the authors use the term GEF when they specifically mean Scd1. This can be a bit confusing to the readers and should be avoided as much as possible.*

The text has been clarified. We only kept the term GEF alone when it was meant in a generic sense (or when referring to *S. cerevisiae* data where there is a single Cdc42 GEF so no possible confusion).

*2.	In the abstract the authors claim that “Ras1 GTPase plays a dual role in localizing and activating the GEF….”. This is a strong statement as we do not know how Ras1 promotes GEF activity, it could function by activating the GEF or facilitating GTPase GEF interaction or by some other unknown means. A more precise wording would be “Ras1 GTPase plays a dual role in localizing the GEF and promoting its activity….”.*

We agree that we did not know how Ras1 promotes the activity of Scd1.This is a similar point to that raised by reviewer 1 in point 6. To address the point, we performed two experiments. We first reasoned that if Ras1 acts through Scd1 to promote Cdc42 activity, *scd1∆* should be epistatic over *ras1∆*. In other words, the levels of Cdc42-GTP at cell tips should be identical in *scd1∆* single mutants and *scd1∆* *ras1∆* double mutants. By contrast, if Ras1 promotes Cdc42-GTP levels by other means, the double mutant should show reduced Cdc42-GTP relative to *scd1∆*, as it does in *scd1+* cells. Measurements of CRIB levels at cell tips of *scd1∆* single mutants and *scd1∆* *ras1∆* double mutants show identical very low values. This is in agreement with epistasis and indeed suggests that Ras1 acts through Scd1. We added this piece of evidence to figure 7E-F. In further support of this idea, in experiments performed in response to point 2 above, we also found that the broad distribution of Cdc42-GTP in cells with uniform Ras1 activity fully depend on Scd1 (please see below). Thus, we feel that the previously unsupported statement that Ras1 promotes activation of Scd1 is now supported by two independent lines of evidence.

*3.	In page 2 last line, Cdc42 localization was also reported by Murray and Johnson 2000, and Coll et al, 2007. These references should be cited.*

The references have been added.

*4.	In page 29 the authors state that “sum projections of five consecutive images are shown”. Do they mean 5 frames along the Z axis? This should be clarified.*

These are sum projections of sequential images in time. This is now clarified.

*5.	In figure 2 the control shown in panel B is not explained wither in the legend or in the text but only explained in the methods. A single sentence explaining the control either in the results or in the legend will help the reader.*

This is now explained in Fig 2B legend. We also explained the use of cell mixtures in the main text.

*6.	Figure 3A, the black arrowheads are not very clear in a grayscale image. Can the authors change the arrowheads to a more obvious color?*

We changed them to orange color.

*--

Reviewer #4: 

In this study, Lamas et. al. study how the spatial localization and activity of the master polarity regulator Cdc42 is controlled in fission yeast. In their study, they modify a number of optogenetic tools for use in fission yeast, an important ‘first’ for this model system. Past studies have shown that Cdc42 activity is controlled by activating factors such as the GEFs Gef1 and Scd1, and also by inactivating factors such as a variety of GAP proteins. In cylindrical fission yeast cells, spatial localization of these proteins helps ensure that active Cdc42 is restricted to the cell ends, which are the sites of polarized growth in fission yeast. If active Cdc42 is mis-localized to the cell sides, cells grow to the wrong shape due to growth in the wrong places. A number of past studies in budding yeast have led to a feedback model, whereby active Cdc42 promotes the recruitment of its activators, namely a scaffold (Bem1, called Scd2 in fission yeast) and a GEF (Cdc24, called Scd1 in fission yeast). Lamas et al demonstrate that Scd1 can be recruited to Cdc42 through two distinct adaptor proteins: Scd2 or the GTPase Ras1. Next, using an optogenetic strategy that leverages the photo-sensitive Cry2-CIB interaction, these authors show that ectopically targeting a constitutively active Cdc42 mutant to the plasma membrane results in recruitment of Cdc42 activators and effectors, which is consistent with a positive feedback mechanism through which Cdc42 promotes its own activation.  Finally, the authors demonstrate that the primary function of the scaffold protein Scd2 is to lead to recruitment of the essential Cdc42 effector Pak1 kinase.

This study has a number of clear strengths.  This study represents the first use of any optogenetic system in fission yeast, at least to my knowledge. The authors do an excellent job of characterizing and validating the robustness of the Cry2-CIB optogenetic system in fission yeast, which will be a strong and broadly applicable tool for the fission yeast community. They also mention in the discussion that other systems did not work in their hands, which could be quite helpful to other labs interested in this approach. While optogenetics has been employed in budding yeast to study the regulation of Cdc42, the new tools developed in this manuscript will enable future studies of Cdc42 in fission yeast, which is comparatively understudied. Additionally, the technical experimental quality of this work is very high, both in the characterization of the optogenetic system and its application to the study of Cdc42 regulation. The experimental design is elegant and clever, and will be
of use for studying many related questions.

Some aspects of the study might limit its overall impact. Primarily, the notion of positive feedback in this system has been studied extensively in budding yeast, and the authors’ conclusions largely fit within the preestablished model. It is worth noting that they are the first to provide strong evidence for this feedback in fission yeast, so the work is likely to be cited widely as evidence for conservation of this feedback, but the conceptual novelty is limited. I also found their experiments and results are largely consistent with positive feedback, but perhaps not as positively conclusive as they have sold it in the text. From my reading of the paper, I felt that the coordination of Ras1 and Scd2 activities represented the most novel finding, but it was not explored in the same depth as the positive feedback model.*

We thank the reviewer for her/his appreciation of our work and constructive comments, which we have addressed in detail below.

*Major comments/concerns
1.	In some parts of the manuscript, the conclusions are seemingly at odds with each other.  For example, the authors state that “Scd1 recruitment to activated Cdc42 fully depends on Scd2”, but the data suggest that Ras1 also functions in recruitment of Scd1 to Cdc42.  Finally, the most novel result in this manuscript is that “Ras1 cooperates with Scd2 for Scd1 recruitment to cell poles”.*

The sentence “Scd1 recruitment to activated Cdc42 fully depends on Scd2” was written in the paragraph on optogenetic assessment of the positive feedback, where Opto-Q61L is used as active Cdc42. In this context, Scd1 recruitment is fully dependent on Scd2. However, in the WT cell, Scd1 recruitment to the cell pole depends not only on Scd2 but also on Ras1, as shown in figure 1. We have now clarified this, by modifying the cited sentence to “these results show that the recruitment of Scd1 to constitutively activated Cdc42 at non-native sites fully depends on Scd2”.

*2.	The authors refer to “dynamic patches formed at the membrane (Figure 1F, Movie 1)” of Cdc42.  These dynamics should be quantified and not just shown in a supplemental movie.  Parameters such as zone formation frequency and duration would be of interest.*

We measured the lifetime of the patches, which clearly shows that the CRIB patches in scd2∆ ras1∆ cells are much shorter-lived than in WT cells. This is added to figure 1G.

*3.	The abstract and introduction should be reworked for clarity and logical flow. For the non-aficionado of Cdc42, the introduction is very hard to follow. The discussion of feedback and past studies in this section makes it even more dense and confusing. There are also a number of typos.*

We appreciate that the field is dense and that much information has to be introduced. In this revised manuscript, we have tried to clarify the introduction and make the logic of arguments more apparent, and hope this is now more digest for the non-specialists.

4.	On Page 8, the authors state that “Because active Cdc42 recruits is own activator Scd1 along with the scaffold protein Scd2 and the effector protein Pak1, this directly establishes the existence of a positive feedback…”. I would not argue that the conclusion has been so strongly proven. For example, the authors have not tested whether WT, or catalytically inactive Cdc42 can also recruit these factors. Therefore, these results could be explained by a mechanism not requiring positive feedback. One option for improvement would be to perform these experiments with the Opto-WT Cdc42 strain for comparison. Given that positive feedback is a central theme of this manuscript, it is important that this result be firmly established in the data.  If WT or a GDP-locked Cdc42 recruit Scd1/Pak1 as efficiently as the GTP locked form, then there is no positive feedback and the result stems from simpler protein-protein interaction mechanisms. In this vein, the experiments in Figure S5B
suggest that not all Cdc42 regulators and effectors are recruited in a manner consistent with positive feedback. The authors are well positioned to determine which proteins (Pak1, Gef1, Scd1, Scd2, Ras1) are recruited by positive feedback and which are not using their optogenetic tools.

In our original submission, we showed that Cdc42-Q61L recruits its GEF Scd1 in a manner dependent on the scaffold Scd2. Therefore, Scd1 does not simply bind to Cdc42-GTP through protein-protein interaction (as also backed by much biochemistry data). In addition, the consequent recruitment of endogenous Cdc42, which also depends on Scd2, is evidence of feedback impacting on the endogenous proteins. By contrast, recruitment of the effector Pak1 or the generic CRIB probe is not dependent on Scd2, as they bind Cdc42-GTP directly. Thus, in the optogenetic setup, Scd1 and Cdc42 are recruited by positive feedback, but Pak1 is recruited directly by Cdc42-Q61L.

To substantiate further the necessity for Cdc42 activation in promoting the feedback, we tested whether Scd2 recruitment depends on Cdc42 activity. For this, we constructed a Cry2-Cdc42-T17N-CaaX∆ allele, yielding Opto-T17N when combined with CIBN-RitC and monitored the recruitment of the Scd2 scaffold, which mediates positive feedback. The Cdc42-T17N allele is predicted to be locked in the GDP form. Opto-T17N did not recruit Scd2, which shows that Scd2 binding to Cdc42 and thus feedback regulation depends on Cdc42 activity. By contrast, the Opto-WT allele did recruit Scd2, at levels that were lower than Opto-Q61L. These data are now shown in Fig 3E and S4B. As discussed in the text, the failure of Opto-T17N to recruit Scd2 shows that scaffold-mediated feedback depends on active Cdc42. Because recruitment and clustering by Cry2 of Opto-WT also leads to some Scd2 recruitment, this indicates Opto-WT becomes activated in these conditions. Clustering-mediated activation has been reported previously for RhoA (Bugaj et al, 2013). However, the activation of Opto-WT is not sufficient to bias the intrinsic cell polarity machinery from cell poles and cells proceeded with polarized growth (Fig. 2G) Although we have not probed this further, these results in combination suggest that feedback regulation may also acts on Opto-WT upon clustering and promote its activation.

*Minor comments/concerns
1.	Some form of data behind Table S1 should be provided.*

Table S1 (previous Table S2) is the data behind Table 1 (previous Table S1). Short of showing the entire reporting table of raw data on the analysed tetrads, we do not have a better way of showing this data.

*2.	In Figure 2G, optogenetic recruitment of GTP locked Cdc42 but not WT leads to cell rounding.  Why is the WT seemingly completely inactive?*

The WT is likely not completely inactive. In fact in our new experiments reported in response to major point 4 above, we have now shown that Opto-WT also leads to some Scd2 recruitment, which depends on Cdc42 activity, thus indicating that clustered Cdc42-WT is somewhat activated. However, in contrast to GTP-locked Cdc42, WT Cdc42 only becomes activated in presence of GEFs and is subject to inactivation by GAPs. Thus, it is likely that WT Cdc42 is only transiently activated and to levels that are not sufficient to compete with the tip-localized Scd1 and Ras1 signals. This is entirely consistent with the observation that endogenous Cdc42 is present in high amounts, but inactive, at cell sides in WT cells (see Bendezu et al, PLoS Biol 2015).

*3.	Why does expression of CRY2PHR-Cdc42Q61L result in nuclear localization of Cdc42 and its regulators to the nucleus? Does this impact any of the experiments?*

In the original paper describing the Cry2-CIBN optogenetic system, the authors claim that all NLS had been deleted resulting in cytosolic localization of CRY2PHR. However, we found some localization of CRY2PHR-mCherry in the nucleus in fission yeast cells. It is thus likely that a cryptic NLS is still present within the CRY2PHR sequence. We note however that CRY2PHR-Cdc42Q61L does not lead to wildtype Cdc42 localization to the nucleus. The nuclear localization of CRIB, which reports on Cdc42-GTP, is present even in WT cells and is thought to be an artefact (see Tatebe et al, Curr Biol 2008). CRY2PHR-Cdc42Q61L however does lead to nuclear localization of Scd2, which we have reported and described in figures 3C and S2B, which has minor dominant-negative effect

*4.	The authors state that “Scd1 recruitment to activated Cdc42 fully depends on Scd2”, but the data suggest that Ras1 also functions in recruitment of Scd1 to Cdc42.  This confusion should be resolved.*

We have clarified this, as explained in response to major point 1 above.

*5.	On Page 10, the authors write that “…the main function of Scd2 is to mediate GEF-PAK complex formation”.  This is confusing given the result that Scd2 is not required for Pak1 recruitment to zones of active Cdc42.  The authors should clarify their hypothesis here.*

Pak1 is a Cdc42 effector that directly binds Cdc42-GTP through its CRIB domain. Therefore, CRY2PHR-Cdc42Q61L (Opto-Q61L) is able to recruit Pak1 independently of Scd2. By contrast, Scd1 requires Scd2 to associate with active Cdc42, as we have shown. Thus, Scd2 is required for Scd1 to join the Cdc42-GTP-Pak1 complex and form a GEF-PAK complex. Because the phenotypes of *scd2∆* can be compensated by directly linking Scd1 to Pak1, this indicates that formation of this GEF-PAK complex is that main function of Scd2.

*6.	The conclusion on P.10 that “lethality suppression was specifically due to feedback restoration and not simply to Scd1 re-localization to cell ends” seems overly strong. As stated above, the results are consistent with positive feedback as well as other mechanisms. Also, the use of Tea1 and For3 are strange controls here (at least for me) because these are different complexes that would not be expected to combine Scd1 and Pak1 in the same complex.  Either the data should be strengthened, or the language softened.*

We rephrased this sentence as follows: Lethality suppression occurred specifically by linking Scd1 to the PAK, but not to another cell tip-localized protein such as Tea1 or another Cdc42 effector such as For3. This suggests that rescue requires proximity of the GEF to the PAK, which cannot be substituted by another Cdc42 effector. The reason for using For3 as a control is that it is a Cdc42 effector, like Pak1. The observation that a For3-Scd1 bridge does not rescue indicates that there is specificity in which effector can be used. The reason for using Tea1 was that Tea1 is prominently localized to cell poles but not in a Cdc42-dependent manner, as it relies on microtubules. The observation that the Tea1-Scd1 bridge does not rescue indicates that the mere recruitment of Scd1 to cell tip (likely through microtubule-dependent delivery) is not sufficient.

*7.	In the conclusions (P.17 and Fig 7J) the authors make a number of assertions that Pak1 localization is critical for relaying positional information to Cdc42, and operates in parallel with Cdc42 positive feedback. These assumptions seem highly testable and relevant to this work.*

We make the inference that Pak1 contains the key localization determinants that define the position of Cdc42 activation in the cell. This is clearly an area of investigation worth pursuing, but this will be by no means easy to test due to the positive feedback and the essentiality of Pak1. Therefore, we feel that this goes beyond the conclusions of this study.
